# Optimization of Incubation Time for Accurate Protein Binding Assessment in Biological Matrices Using Equilibrium Dialysis

**DOI:** 10.2174/0118723128441303260129080416

**Published:** 2026-03-18

**Authors:** Jay Prakash Jain, Jianxia Shi, Atul Rathore, Austin Li, Chunyan Han, Xingjin Jiao, Richard Zang

**Affiliations:** 1 DMPK and Clinical Pharmacology, IDEAYA Biosciences, South San Francisco, CA, USA;; 2 DMPK Division, Pharmaron Inc., Beijing, China

**Keywords:** Protein Binding (PB), Rapid Equilibrium Dialysis (RED), HTDialysis (HTD), equilibrium time, % unbound, tumor binding

## Abstract

**Introduction:**

Accurate % unbound estimation in protein binding assays in drug discovery is critical for understanding Pharmacokinetics (PK), Pharmacodynamics (PD), and safety assessments. Short incubation times (2-6 hours) in Equilibrium Dialysis (ED) compromise the determination of the % unbound. Incubation times were evaluated in ED to obtain reliable estimates of % unbound across diverse biological matrices and compound types.

**Methods:**

A time-course of 2-24 hours for 17 marketed compounds with varying physicochemical properties was conducted using RED and HTD devices across human plasma, tumor cell lines, 10% FBS, and rodent tissues to determine % unbound values.

**Results:**

Compounds exhibited unbound fractions ranging from <1% to >70% and generally correlated with LogD. Incubation times <6 hours underestimated % unbound (up to 6-fold), particularly for highly bound compounds. Equilibrium was typically reached by 16–24 hours in all matrices. A <20% increase was exhibited in % unbound beyond 16 hours (except lapatinib). HTD yielded a marginally faster equilibrium than RED.

**Discussion:**

Short incubation times (2–6 hours) in equilibrium dialysis often underestimate the % unbound, particularly for highly bound compounds. Extending incubation to ≥16 hours achieved equilibrium across plasma, tissues, and cell lines, yielding more reliable and consistent % unbound data. This standardized approach improves the PK/PD assessment and compound prioritization in early drug discovery.

**Conclusion:**

The usual 2-6 hours of incubation in ED underestimates % unbound and may be inadequate for compound prioritization. We recommend ≥16 hours of incubation for protein binding assessment in early drug discovery.

## 1 INTRODUCTION

Drug Protein Binding (PB) in biological matrices, such as plasma, is a simple yet intricate phenomenon, particularly when quantifying compounds with low unbound fractions. While Plasma PB (PPB) itself is not typically optimized during drug development [1-3], accurately assessing PB in bioassay media, plasma, tissues, or disease-specific cell lines is critical for extrapolating *in vitro* potency, *in vitro–in vivo* correlations (IVIVCs), dose projections for animal and human studies, and estimating safety margins.

The accurate determination of the % unbound of a drug candidate is crucial in the context of the free drug hypothesis [4-6]. At steady-state, unbound drug concentration in tissues is assumed to be in equilibrium with plasma; however, concentrations in tissues could be higher or lower depending on active transport mechanisms [7]. Therefore, it is crucial to assess PB values in multiple biological matrices (tissues, cell lines, and plasma) and animal species for a comprehensive understanding of tissue and plasma distribution. In oncology, PB studies of investigational drugs in tumor cell lines and tissues may provide insights into pharmacokinetic/pharmacodynamic (PK/PD) relationships and support the optimization of therapeutic strategies for disease-specific targets [8]. Tumor cell lines mimic a complex tumor microenvironment, which has high protein content, an altered extracellular matrix, and variable transporter expression profiles. These factors can affect drug binding, drug retention, and cellular drug availability. Likewise, PB in animal tissues, such as liver and brain, differs from plasma due to its unique protein compositions and physiological barriers. Measuring the % unbound in such matrices enables more accurate estimation of intracellular drug exposure, interpretation of preclinical PK data, and prediction of human tissue distribution.

Among the methods used to determine PB in drug discovery, the “workhorse assay” for PB is equilibrium dialysis, performed with Rapid-Equilibrium Dialysis (RED) or High-throughput Dialysis (HTD) devices using undiluted plasma [9]. Given the increasing demand for faster data turnaround in drug-discovery settings and also considering the logistical challenge of total assay run time, equilibrium dialysis for PB estimations performed with short incubation times, such as 2 to 6 hours, is not uncommon [9-11]. An inadequate incubation time that does not ensure complete dialysis equilibrium will not yield accurate % unbound values.

Therefore, it is crucial to determine the appropriate incubation time for dialysis equilibrium in an extended time-course PB study. This study conducted time-course PB assays using diverse biological matrices, including human plasma, cell culture medium containing 10% Fetal Bovine Serum (FBS), and rat brain homogenate, with 15 marketed compounds representing structurally diverse therapeutic classes and varying lipophilicity. Incubation time-course data from these first 15 compounds were later used to study the PB behavior of two additional highly lipophilic drugs, nelfinavir and ritonavir, in two matrices– human/mouse tumor cell lines and mouse tissues. These experiments aim to standardize current PB assays using ED and provide relevant data to drug discovery teams to support their PB assessment strategies.

## 2 MATERIALS AND METHODS

### 2.1 Materials

Human plasma, FBS buffer, rat brain homogenate, 17 marketed drugs (axitinib, clozapine, dasatinib, erlotinib, fluphenazine, imatinib, ketoconazole, lapatinib, sildenafil, sunitinib, tolbutamide, dexamethasone, fluconazole, propranolol, sulfamethoxazole, nelfinavir, and ritonavir), internal standards (ISs), mouse tissues (including brain, intestine, liver, lung, adipose, muscle, skin, and plasma), phosphate-buffered saline (PBS), a RED device, RED Device Reusable Base Plate (Thermo SCIENTIFIC, Prod #89811), RED Device Inserts (Thermo SCIENTIFIC, Prod #89809), and an HTD device (HTDialysis LLC, Gales Ferry, CT) were used in this study. Mouse tissues (brain, intestine, liver, lung, adipose, muscle, and skin) were obtained from female BALB/c mice (8-10 weeks old, 17-25 g), with 30 animals used for brain, intestine, liver, and lung tissues, and 50 animals used for adipose, muscle, and skin tissues. Rat brain tissue was obtained from male Wistar Han rats (6-7 weeks old, 150-250 g; n = 50). Human cell lines CAMA-1 (ATCC HTB-21), JIMT-1 (Cobioer CBP60378), SW837 (ATCC CCL-235), NCI-H650 (ATCC CRL-5835), and NCI-H2122 (ATCC CRL-5985), and murine cell lines CT26.WT (ATCC CRL-2638) and B16-F10 (ATCC CRL-6475) were obtained from accredited cell banks. All lines were authenticated, confirmed mycoplasma-free, and cultured under supplier-recommended conditions. Frozen human plasma (pooled from 195 male and female donors) was obtained from healthy adult (18 to 55 years old) Asian volunteers at Liaocheng Second People’s Hospital (Liaocheng, Shandong, China) under ethical approval from the Ethics Committee of Liaocheng Second People’s Hospital (Approval No. KB2023-Ethical Review Report-51-01). All procedures complied with the Declaration of Helsinki (2013 revision), and written informed consent was obtained from all donors prior to sample collection. LC-MS/MS grade solvents were used for analysis. All animal-related procedures for tissue collection were conducted in compliance with current regulations and approved by the Institutional Animal Care and Use Committee (IACUC) of SPF (Beijing) Biotechnology Co., Ltd., Beijing, People’s Republic of China (Approval Nos.: AWE2024-12-27 and AWE2024-12-2702), adhering to the 3Rs principle and the ARRIVE guidelines for ethical reporting of animal research.

### 2.2 Methods

#### 2.2.1 Drug-selection Criteria

Seventeen marketed compounds were selected to represent different drug classes with varying acidic/basic properties, LogD_7.4_, and PB characteristics (Table **1**). Of these, ritonavir and nelfinavir were highly lipophilic drugs, having high and very high PB characteristics, respectively.

#### 2.2.2 Preparation of Matrices

The study was conducted in 2 parts. The first part determined the optimal incubation time for 15 marketed drugs (axitinib, clozapine, dasatinib, erlotinib, fluphenazine, imatinib, ketoconazole, lapatinib, sildenafil, sunitinib, tolbutamide, dexamethasone, fluconazole, propranolol, and sulfamethoxazole) using human plasma, cell culture medium containing 10% FBS, and rat brain homogenate. In the second part of the study, the PB properties of nelfinavir and ritonavir were determined using tumor cell lines and mouse tissues (brain, intestine, liver, lungs, adipose, muscle, skin, and plasma). The animals were housed in groups of 2-3 animals per cage in a climate-controlled rodent facility with a 12 hours light/dark cycle. The animals had ad libitum access to water and food. Animals were euthanized in an anesthesia chamber using carbon dioxide (CO_2_) at a 50% gas exchange rate per minute for 5 minutes. Following confirmation of respiratory cessation, physical methods (cervical dislocation, cardiac puncture, or exsanguination) were applied to ensure death. Tumors and tissues were collected aseptically using sterile scissors and forceps, immediately placed into pre-labeled cryovials, snap-frozen in liquid nitrogen, and stored at -80°C until further processing.

Rat brain homogenates were prepared by adding 3 volumes (mL) of PBS (pH 7.4) to 1 volume (g) of whole brain tissue, followed by homogenization using a tissue homogenizer. Mouse tissue and tumor homogenates were prepared using 100 mM sodium phosphate and 150 mM NaCl buffer (PBS), with tissue-to-buffer ratios of 1:3 for brain, liver, adipose, and tumor tissues; 1:7 for skin, intestine, and lung; and 1:9 for muscle. Homogenization was performed under low-temperature conditions. All homogenates were stored at -80°C and thawed at room temperature immediately before use.

Pooled human plasma (pH adjusted) was obtained from healthy Asian volunteers from a domestic hospital under ethical approvals. To prepare 10% FBS, the frozen FBS stock was thawed overnight at 4°C. Cell culture medium containing 10% FBS was prepared by mixing FBS with 1% penicillin-streptomycin solution in Roswell Park Memorial Institute 1640 (RPMI1640) medium (HyClone, USA). RMPI640 was used as it is a common medium employed for in-house cell culture.

### 2.3 Determination of PB using Equilibrium Dialysis

An aliquot of the compound dissolved in Dimethyl Sulfoxide (DMSO) stock solution was used to prepare 200 µM of experimental working solutions. The experimental working solutions were diluted 1:100 in the matrix used for PB. The final compound concentration for equilibrium dialysis experiments was 1 µM in 0.5% DMSO. All samples were vortexed thoroughly before use.

Assays to optimize equilibration time were conducted in human plasma, 10% FBS, and rat brain homogenate, with 15 compounds at different time points over a 24-hour period. After optimizing the incubation time, the PB characteristics of nelfinavir and ritonavir were assessed at 6 and 16 hours of incubation time using tumor cell lines and mouse tissue homogenates.

Equilibrium dialysis was performed using RED (ThermoFisher Scientific, San Francisco, USA) and HTD (HTDialysis LLC, Gales Ferry, CT, USA) devices [12] across various biological matrices. Human plasma and 10% FBS, generally serving as common matrices for routine PB experiments, were evaluated using both RED and HTD devices to enable direct comparisons. Studies in other matrices were performed using either RED or HTD devices.

#### 2.3.1 RED Device

RED is the most common method for assessing protein binding, and its methodology is well established [5, 11]. Briefly, RED device inserts were placed with their open ends up into the wells of the base plate. Spiked matrices (300 μL) to standardize extended incubation times were added into the sample (donor) chamber, which was indicated by the red-marked ring. 100 mM of sodium phosphate and 150 mM NaCl (PBS) (500 μL) were added to the buffer (receiver) chamber. Two replicates were performed for each experiment. The unit was covered with sealing tape and incubated at 37°C (with 5% CO_2_) while shaking at 300 rpm. In the first part of this study, aliquots of the 15 compounds were collected at 2, 6, 16, and 24 hours. For the subsequent part of the study, aliquots of nelfinavir and ritonavir were only collected at 6 and 16 hours. Each assessment was performed in duplicate.

#### 2.3.2 HTD Device

Dialysis membranes with a molecular weight cut-off of 12–14 kDa (HTDialysis LLC, Gales Ferry, CT) were soaked in ultrapure water for 60 min to separate the strips and then soaked in 20% ethanol and dialysis buffer for 20 min each. The dialysis apparatus was assembled following the manufacturer’s instructions [12]. Each cell was filled with 120 μL of the spiked matrices and dialyzed against an equal volume of dialysis buffer (PBS). Two replicates were performed for each assay. The dialysis plate was sealed and incubated at 37°C with 5% CO_2_ while shaking at100 rpm. Sampling was conducted at 2, 6, 16, and 24 hours for the 15 compounds, and at 6 and 16 hours for nelfinavir and ritonavir. Each assessment was performed in duplicate.

#### 2.3.3 Sample Processing

At each time point, a 50 µL aliquot was taken from both the matrix and buffer chambers of each device and transferred to separate wells of a 96-well plate. The 0 min time point sample was prepared by transferring 50 µL of the spiked tumor tissue sample to 50 μL of blank buffer and then adding 300–400 μL of quench solution at room temperature. For matrix match, 50 μL of the blank matrix was added to each buffer sample, and an equal volume of buffer was supplemented to the matrix sample. Samples were quenched at room temperature with acetonitrile containing 3% formic acid with the internal standards (ISs, 100 nM alprazolam, 500 nM labetalol, and 2 μM ketoprofen) to precipitate proteins. Samples in the plate were vortexed for 5 min and centrifuged at 3,220 ×g for 30 min at 4°C. A volume of 100 μL of the supernatant was transferred to a new 96-well plate with 100 μL of water for LC-MS/MS.

### 2.4 LC-MS/MS

An LC system (Shimadzu) coupled to a Triple Quad 5500+ mass spectrometer (SCIEX, Canada) was used. The detection and quantification of test compounds were conducted using an XSelect HSS T3 2.5 µm (2.1 × 50 mm) column attached to a pre-guard column. The mobile phases, consisting of water with 0.1% formic acid (A) and acetonitrile with 0.1% formic acid (B), were delivered to the column using a linear gradient at a flow rate of 0.6 mL/min. MS data for all compounds, except fluconazole, were acquired in the positive electrospray ionization mode. The multiple reaction monitoring mode was used for the qualitative and semi-quantitative analyses of the test compounds. The concentration of the test compounds was quantified using the area ratios, *i.e*., analyte peak area/IS peak area. Data acquisition and analysis were performed using the Analyst software version 1.7.2.

### 2.5 Determination of the Physicochemical Properties of the Test Compounds

#### 2.5.1 Distribution Coefficient at pH 7.4 (Log D7.4)

LogD_7.4_ values were determined in-house using the conventional shake-flask method [13] for all compounds except for nelfinavir and ritonavir, for which values were obtained from an online DrugBank repository (https: //go.drugbank.com) [14]. Briefly, working solutions of each compound were mixed with PBS (pH 7.4) saturated with 1-octanol in a 96-well plate, followed by the addition of 1-octanol-saturated PBS (pH 7.4). The plates were shaken at 25°C at 1,100 rpm for 1 hour. The samples were then centrifuged at 25°C at 20,000 ×g for 20 min to separate the phases. Aliquots (5-50 µL) were taken from the upper or lower phase, followed by a dilution of water and acetonitrile (1:1 v/v). The dilution factor was adjusted based on the LogD_7.4_ value and LC-MS/MS signal response. The diluted samples were transferredinto a new 96-well plate and analyzed using the described LC-MS/MS method. The MS peak area, dilution factor, and injection volume of each phase were used to calculate the LogD_7.4_ values.

#### 2.5.2 Acid-base Dissociation Constant (pKa)

Fast-ultraviolet (UV) or pH-metric methods were used to determine the pKa values of compounds [15-17]. The pKa values of fluconazole, sildenafil, nelfinavir, and ritonavir were obtained from an online DrugBank repository (https://go.drugbank.com). If changes in the ionization state of the compounds led to an observable change in UV absorbance at wavelengths ≥240 nm, the fast-UV method was used to determine the pKa of compounds, and if not, the pH-metric method was used. Depending on the compound's solubility, the pKa was determined with or without a cosolvent. Methanol was usually used as a cosolvent at three different weight ratios. For the fast-UV method, following the slow addition of different acid or base titrants, spectral data at different pH values were collected, and turbidity was determined by spectrometry. Similarly, for the pH-metric method, with the addition of different acid and base titrants, the titration curves by pH were collected, and the turbidity was detected by using a turbidity sensor. The titration range of pH, determined by using both UV and pH-metric methods, was 2–12. The titration data were obtained following proper titration, and the pKa values were obtained using the Yasuda-Shedlovsky extrapolation method [17, 18].

### 2.6 Data Analysis

The concentrations of the compounds were determined from the peak area ratios in the receiver (PBS buffer) and the donor compartments of the equilibrium dialysis devices. The % unbound of all compounds (% unbound or (% unbound_d _for diluted samples)) was determined by using Eq. (1).


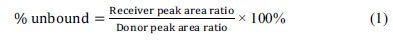


The values obtained were for the diluted percentages of the unbound compounds, and these were corrected by applying the dilution factor for the actual % unbound values as presented in Eq. (2). 


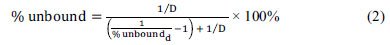


The percentage bound in each of the matrices were calculated using Eq. (3).


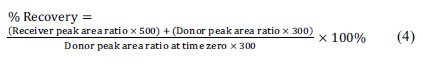


The device recovery and the compound stability were each determined using Eqs. (4 and 5), respectively.


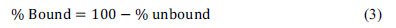



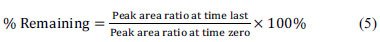


The peak area ratio in the buffer (receiver) chamber indicates the concentration of the % unbound, and the peak area ratio in the matrix chamber (donor) indicates the concentrations of both the unbound and bound percentages.

## 3 RESULTS

### 3.1 Compounds and their Physicochemical Properties

The basic physicochemical properties and structures of all compounds used in the assay are presented in Table **1**, representing a range of lipophilicity, ionization states, and protein binding characteristics**.** For the plasma protein binding incubation time optimization, a total of 15 compounds were selected, comprising of 12 basic, 2 acidic, and 1 neutral compounds. These compounds ranged from hydrophilic (LogD_7.4_, –0.62) to significantly lipophilic (LogD_7.4_, 3.81). The broad range of physicochemical properties allowed a comprehensive assessment of how incubation time to equilibrium affected measured plasma protein binding values across different classes of compounds. Based on the published results [19-21], these compounds represented a spectrum ranging from highly bound (>99% bound in human plasma) to weakly bound (nearly 100% unbound). As shown in Fig. (**1**), a general trend of correlation between LogD_7.4 _*vs.* % bound was observed, which was consistent with the consensus that lipophilicity is a determining factor for % unbound [22, 23]. Following the initial plasma incubation time optimization experiments, the high lipophilicity compounds nelfinavir and ritonavir were selected for further evaluation of optimal incubation time in tumor cell lines and tissue-binding studies.

### 3.2 Stability and Recovery of Compounds

To validate the % unbound results, the stability of each compound was assessed in study matrices and were based on the percentage of compound remaining over the study’s duration. Fifteen compounds in the study were found to be stable in human plasma, 10% FBS, and rat brain homogenate, with stability ranging from 76% to 119% (data on file). Nelfinavir and ritonavir were stable in the range of

70%-110% in tumor cell lines (data on file), whereas nelfinavir was found to have the least amount remaining (31%-60%) in adipose, muscle, and skin tissues of mice (data on file). In other matrices, both compounds showed stability in the range of 70%-116%. Additionally, the compound recovery for different durations of incubation was assessed and found to be consistent across time points. Although recovery is unlikely to impact the % unbound [24], it was found to be in the reasonable range of 40%–119% for the test compounds when RED and HTD devices were used for human plasma, 10% FBS, and rat brain homogenate. Recovery for nelfinavir was >74% in all matrices except adipose, muscle, and skin tissues (data on file). No specific trend of recovery was noted in this limited data set with respect to % unbound and LogD_7.4_ for the 15 compounds (Fig. **1**).

### 3.3 Assessment of % Unbound and Equilibrium Time

Time profiles of PB in human plasma, 10% FBS, and rat brain homogenate for the 15 compounds in RED and HTD devices are presented in Fig. (**2**) and Supplemental Tables **1**-**3**. Except for lapatinib at some time points in both the RED and HTD devices, all compounds yielded measurable values for % unbound. The % unbound in all matrices at the 24-hour equilibrium time represented a wide range from <1% to nearly 100%. In 10% FBS, except for lapatinib, all compounds had a % unbound of >10%, whereas in the plasma and rat brain homogenate, most compounds exhibited a value of <10%. Fig. (**2A**–**H**) demonstrate that in human plasma and brain homogenate, over 80% of compounds had not reached equilibrium by 6 hours. The % unbound at 2 hours was considerably lower than the value obtained for the 24-hour incubation (assumed equilibrium), and the difference between the % unbound values was high. The trend was consistent for highly bound (<10% unbound) and for moderately to weakly bound (>10% unbound) compounds. By 16 hours, nearly all of the compounds had reached equilibrium, as indicated by the plateau in the % unbound values observed at 24 hours. In contrast, over 85% of compounds in 10% FBS reached equilibrium by 6 hours, independent of the device type (Fig. **2E**-**F**). The time course scatter plots for human plasma, 10% FBS, and rat brain homogenate show the stability of the % unbound over time, with the % unbound time point plotted against the absolute % unbound at 24 hours (Fig. **3A**-**H**). Compared with the highly variable early phase at 2 hours, only 3 of the 14 quantifiable compounds (lapatinib excluded due to BLQ) attained near-equilibrium by 6 hours, while strongly bound drugs continued to show variability. At 16 hours, consistent equilibrium was achieved for nearly all quantifiable compounds (14 of 15; lapatinib excluded due to BLQ values). In the 10% FBS matrix, the % unbound values at 6 hours closely matched those at 16 hours, indicating that equilibrium was established by 6 hours and maintained thereafter (Fig. **3E**-**F**). These results were consistent across compounds and between both the devices.

Relatively faster equilibrium was attained when using the HTD device compared with that attained using the RED device, especially for human plasma and for compounds that were <10% unbound (Figs. **2** and **3**). For example, the % unbound at 6 and 16 hours were comparable (*i.e*., <20% difference) for 7 compounds obtained using the HTD device, compared with 2 compounds obtained using the RED device (Supplemental Table **1**).

### 3.4 Assessment of % Unbound in Tumor Cell Lines and Mouse Tissues

Using the optimized incubation times, the tumor-binding properties of nelfinavir and ritonavir were evaluated across breast, colorectal, lung, and B16F10 tumor cell lines. The evaluation was performed at two incubation time points (6 hours and 16 hours) as shown in Supplemental Table **4**. The results demonstrated consistent and comparable binding values across different tumor cell lines. Nelfinavir demonstrated extremely low % unbound values across all tumor models at 6 hours, ranging from 0.06% to 0.11%, which increased by 2- to 3-fold (0.13% to 0.23%) after 16 hours of incubation (Supplemental Table **4**). The extended incubation revealed significant increase in the % unbound of tested compounds in colorectal models (*e.g*., SW837, where the % unbound increased from 0.08% to 0.23%, a ratio of 2.85) and in breast models (*e.g*., CAMA-1, where the % unbound increased from 0.06% to 0.18%, a ratio of 2.95), highlighting the importance of prolonged incubation for capturing time-dependent changes in highly bound compounds such as nelfinavir.

Ritonavir also displayed high PB across tumor cell lines, with % unboundvalues consistently higher than Nelfinavir’s, ranging from 1.12% to 1.77% at 6 hours (Supplemental Table **4**). Comparable values of Ritonavir were obtained between 6 and 16 hours, indicating a faster attainment of binding equilibrium (Fig. **4A** and **B**). The 16-hour incubation revealed subtle time-dependent changes, such as slight increase in the % unbound in breast model JIMT-1 (from 1.14% to 1.23%) and decreases in lung model NCI-H2122 (from 1.18% to 0.94%).

The PB properties of nelfinavir and ritonavir were further evaluated in various mouse tissues, including the brain, intestine, liver, lungs, adipose, muscle, skin, and also in plasma over the extended durations of 6 and 16 hours (Fig. **5A, 5B,** and Supplemental Table **5**). The results revealed tissue-specific binding patterns, with consistent trends and time-dependent changes that were more pronounced with extended incubation. Nelfinavir exhibited exceptionally low % unbound values across all tissues, ranging from 0.03% in the liver to 0.13% in adipose tissues at 6 hours. These findings reflect its strong binding to tissues, with the % bound consistently exceeding 99.8% in all tissues. The 16-hour incubation period revealed substantial increase in the % unbound for nelfinavir, particularly in adipose tissues (from 0.13% to 0.39%, a ratio of 2.89), and the liver (from 0.03% to 0.16%, a ratio of 4.76). Even in plasma, the % unbound nearly doubled (0.06% to 0.11%, a ratio of 1.87). These findings highlight the persistent and robust binding of nelfinavir across tissues, even under extended incubation conditions.

Ritonavir showed higher % unbound values across all tissues relative to nelfinavir. After 6 hours, the % unbound ranged from 0.07% in the liver to 2.83% in adipose tissues. At 16 hours, the % unbound increased in all tissues except in skin, reflecting better equilibrium over time. In adipose tissues and plasma, the % unbound increased from 0.07% to 0.27% (a ratio of 3.72), and from 0.18% to 0.38% (a ratio of 2.09), respectively. In brain tissues, the % unbound increased from 1.13% to 1.26% (a ratio of 1.12), suggesting minor time-dependent changes in the binding profile of ritonavir. In both nelfinavir and ritonavir, values of the % unbound for the skin were similar between 6 and 16 hours, indicating early attainment of equilibrium.

## 4 DISCUSSION

Rapid, high‑throughput screening of compounds in ADME assays is essential in drug discovery. PB measurements are commonly determined during primary screening but may not be re-evaluated until later stages during characterization for regulatory purposes, for which several techniques have been validated recently for robust and relevant estimation of % unbound for highly bound and challenging compounds [7, 25-31]. The accurate determination of the % unbound across matrices, particularly in plasma, tissue homogenates, and relevant *in vitro* media, is therefore critical early-on because % unbound values are used in the interpretation of *in vitro* potency, *in vitro*–*in vivo* correlations (IVIVC), dose projections for animals and humans, and safety margins, especially for highly bound compounds [32, 33].

When % unbound is measured before achieving dialysis equilibrium by RED and HTD devices, the true % unbound value may be underestimated by several fold. Such underestimation of % unbound values in assay media can overestimate apparent potency, while underestimating the % unbound in plasma can bias hepatic clearance predictions downward or upward, depending on the scaling context [24, 34]. For meaningful cross‑species comparisons and efficacious‑dose projections, total drug concentrations (*in vitro* or *in vivo*) must be corrected for % unbound; imprecise % unbound measurements can misdirect lead optimization [29]. Kinetic modeling approaches to estimate the optimum equilibrium time are available [35] however given the impracticality of optimizing the equilibrium time for each compound during early discovery, characterizing the time required for most compounds to reach dialysis equilibrium and implementing it as the standardized assay condition is essential to reduce the risk of % unbound underestimation and to enhance data consistency across compounds, facilitating reliable characterization of lead candidates in drug discovery. In this work, we evaluated PB over time for a panel of marketed compounds spanning diverse physicochemical properties. We profiled multiple matrices that are used routinely in discovery (human plasma, 10% FBS, rat brain homogenate), and extended the assessment to tumor cell lines and selected mouse tissues. Rat brain homogenate was included because its % unbound has been reported to approximate human brain % unbound, enabling a translational utility without interspecies correction [7, 9]. Among the physicochemical descriptors, LogD_7.4_ (lipophilicity at pH 7.4) tracked as expected with the extent of PB. Therefore, nelfinavir and ritonavir were chosen for tumor and tissue binding experiments due to their high LogD values.

Both RED and HTD devices meet high‑throughput needs and are widely adopted for PB estimation. Prior reports have suggested that 2–6 hours may be sufficient to reach equilibrium [10-12]. The results in these studies were limited by inadequate incubation times of 6 hours, or the lack of intermediate time points between 6 and 24 hours, and other methodological limitations, such as comparisons made based on the % bound values [10, 11]. Therefore, a plateau curve indicative of true equilibrium and % unbound could not be confirmed. This approach to compare the % bound values could be misleading, particularly for highly bound compounds, where small changes in % bound can mask significant differences in % unbound [10, 11], which is a more relevant parameter. For example, a change from 99.5% to 99% bound appears to represent only a 0.5% or negligible difference, which corresponds to a 2-fold change in the % unbound values (0.5% to 1%).

The matrix composition affected the apparent rate of reaching equilibrium. In 10% FBS, many compounds displayed a higher % unbound than in plasma or other tissues, which can be attributed to the lower protein content and less nonspecific binding. For compounds with <10% unbound in plasma at 2 hours, the % unbound values were on average ~2.5-fold and up to ~6-fold (for imatinib) underestimated compared to those at equilibrium, irrespective of using a RED or HTD device. At the 6-hour incubation, it was still, on average, ~50% and up to a ~2.5-fold underestimation of the % unbound values in the RED device, and ~25% and up to a ~1.7-fold underestimation of the % unbound values in the HTD device. At 16 hours, a difference of <10% in % unbound was noted in both devices. A slightly faster equilibrium was achieved when using the HTD device; however, significant underestimation of % unbound can still occur with an incubation time of ≤6 hours. For compounds with % unboundvalues of >10%,the errors were smaller at the shorter incubation times, but in both RED and HTD devices, % unboundvalues were underestimated up to ~25% for equilibration times at ≤6 hours. As the dialysis incubation time was increased, the measured % unbound values gradually increased until equilibrium was attained. Interestingly, for sunitinib in human plasma, the % unbound values measured at 24 hours using the RED and HTD devices were 8%–9%, which were nearly 2-fold higher than the reported value of 5% [36]. These findings suggest that a longer incubation time (>16 hours) is necessary to maximize the likelihood of achieving dialysis equilibrium. However, due to limited data, definitive correlations between specific compound characteristics and optimal incubation times could not be established.

In 10% FBS, most compounds reached a % unbound plateau by 6 hours, except fluphenazine and lapatinib, which exhibited the lowest % unbound. At 2 hours, the % unbound was generally underpredicted by 2- to 3-fold. These findings suggest that for compounds having a % unbound value of <30% in 10% FBS, it is recommended to use an incubation time of 6–16 hours, consistent with that used for the plasma matrix.

The recently and commonly used diluted plasma approach, in which plasma is diluted up to 20-fold with PBS (pH 7.4) for RED or HTD systems, has yielded quantifiable and reliable % unbound values for highly bound compounds [7]. The equilibrium time may be shorter for matrices such as 10% FBS, which have lower protein concentrations; however, if the % unbound of the compound remains <30%, a longer equilibration time may be required. The initial determination of LogD7.4 is a critical factor for guiding this decision. The diluted plasma approach was not adopted in this study to evaluate compounds such as lapatinib, where a quantifiable concentration of the % unbound could not be acquired at some of the time points. PB studies in both tumor cell lines and brain tissue homogenates are commonly conducted to determine the brain-to-plasma unbound concentration ratio (Kp, uu)[8]. An equilibration time of 16 or 24 hours is suggested for these matrices, as well as for tissues that generally have a higher protein content and a % unbound value than that in plasma_. _ Despite a limited sample size (n=15), no notable differences or trends were observed in PB and physicochemical properties such as molecular weight, LogD, acidity, basicity, zwitterionic state, or neutral nature in this study. This could be due to the possibility that compounds may bind to different plasma protein components with different binding affinities [9, 37]. All test compounds used in this study were stable and had good recoveries; therefore, unstable or poorly recovering compounds may require additional consideration during their % unbound determination. In a separate experiment, the PB of nelfinavir and ritonavir was assessed in tumor cell lines at 6 and 16 hours. Two distinct cell line variants of each tumor type were selected for this study to capture intratumoral heterogeneity and clinically relevant differences. B16F10 is a metastatic murine melanoma model frequently used for metastasis and immunotherapy-related preclinical studies. For nelfinavir, the extended incubation period confirmed its strong binding, with extremely low % unbound values at 6 hours (0.06% to 0.11%) and a 2- to 3-fold increase by 16 hours (0.13% to 0.23%) across tumor models, demonstrating the importance of prolonged incubation times for capturing the time-dependent binding dynamics in highly bound compounds. Ritonavir also displayed high PB across tumor models, with comparable % unbound values between 6 and 16 hours, indicating faster equilibrium attainment. The longer incubation period (*i.e*., 16 hours) revealed subtle changes in binding and incremental increases in % unbound compared with the shorter incubations. These findings emphasize the critical role of extended incubation periods in detecting time-dependent changes in highly bound compounds.

The PB properties of both nelfinavir and ritonavir were further evaluated in mouse tissues, including brain, intestine, liver, lung, adipose, muscle, skin, and plasma, exhibiting consistent, tissue-specific binding trends. Nelfinavir displayed exceptionally low % unbound values, reflecting its strong binding to tissues (>99.8% bound). The 16-hour incubation led to substantial fold increases in % unbound, particularly in liver (~4.8-fold) and adipose (~2.9-fold). Ritonavir showed higher % unbound values across all tissues, ranging from 0.07% in the liver to 2.83% in adipose at 6 hours. In comparison to 6 hours, the % unbound increased in all tissues (except skin) at 16 hours incubation time, including a ~3.7-fold rise in liver, and a ~1.4-fold increase in adipose tissue, indicating better equilibrium. These findings underscore the importance of extended incubation times in assessing protein–drug interactions and support the use of longer incubation periods to enhance the predictive accuracy of drug behavior *in vivo*.

## 5 STUDY LIMITATIONS

The study was limited by the number of compounds representing different physicochemical classes therefore, a trend analysis cannot be performed for PB. Furthermore, the PB for the tool compound was not assessed using additional techniques such as flux dialysis, diluted plasma, presaturation method, ultracentrifugation, *etc*. to confirm % unbound using an orthogonal assay.

## CONCLUSION

Accurate determination of the % unbound of a compound in plasma or other biological matrices is critical for characterizing compounds in drug discovery. Insufficient equilibrium times during PB assessment can result in significant errors in estimating % unbound values, which may subsequently impact the interpretation of *in vitro* potency, IVIVCs, dose projections for animal and human use, and the estimation of safety margins for compounds. These errors can ultimately affect the rank order or prioritization of compounds. Based on the current study, an equilibrium time of at least 16 hours is recommended for PB by dialysis using the RED or HTD devices across plasma, tissues, and disease-relevant matrices at relevant concentrations. This approach minimizes underestimation of % unbound values and provides consistent data across compounds, supporting the accurate characterization of lead candidates during drug discovery.

## Figures and Tables

**Fig. (1):**
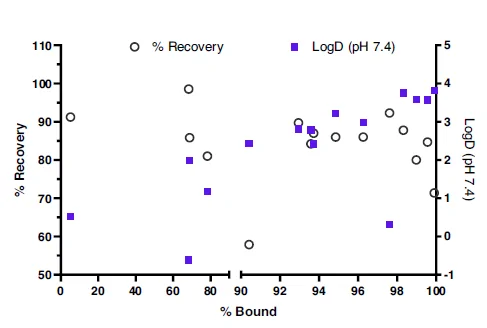
Correlation of recovery and LogD with respect to % bound following a 24-hour incubation in human plasma for 15 compounds using the RED device.

**Fig. (2):**
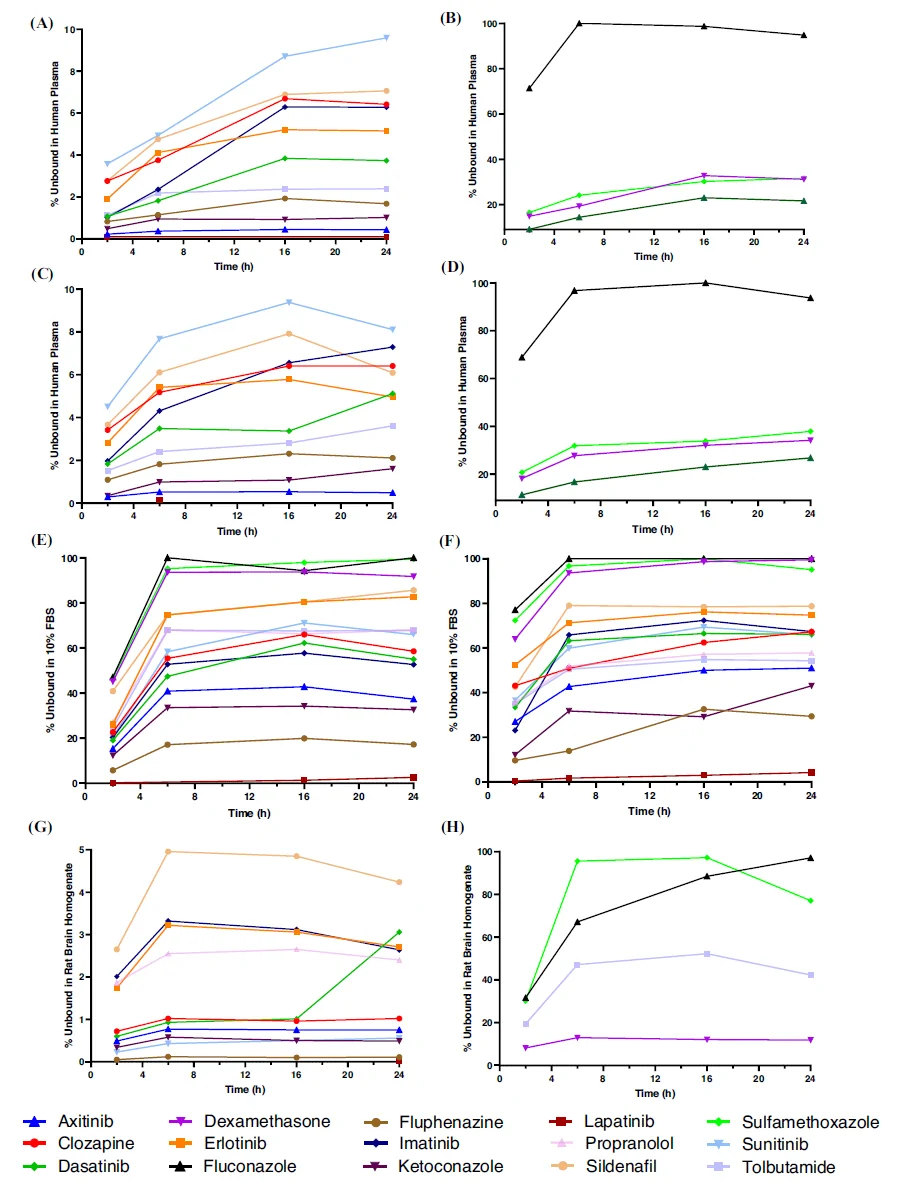
% unbound time profile up to 24 hours (**A**) using the RED device for <10% unbound in human plasma; (**B**) using the RED device for >10% unbound in human plasma; (**C**) using the HTD device for <10% unbound in human plasma; (**D**) using the HTD device for >10% unbound in human plasma; (**E**) using the RED device in 10% FBS; (**F**) using the HTD device in 10% FBS; (**G**) using the HTD device for <10% unbound in rat brain homogenate; (**H**) using the HTD device for >10% unbound in rat brain homogenate.

**Fig. (3):**
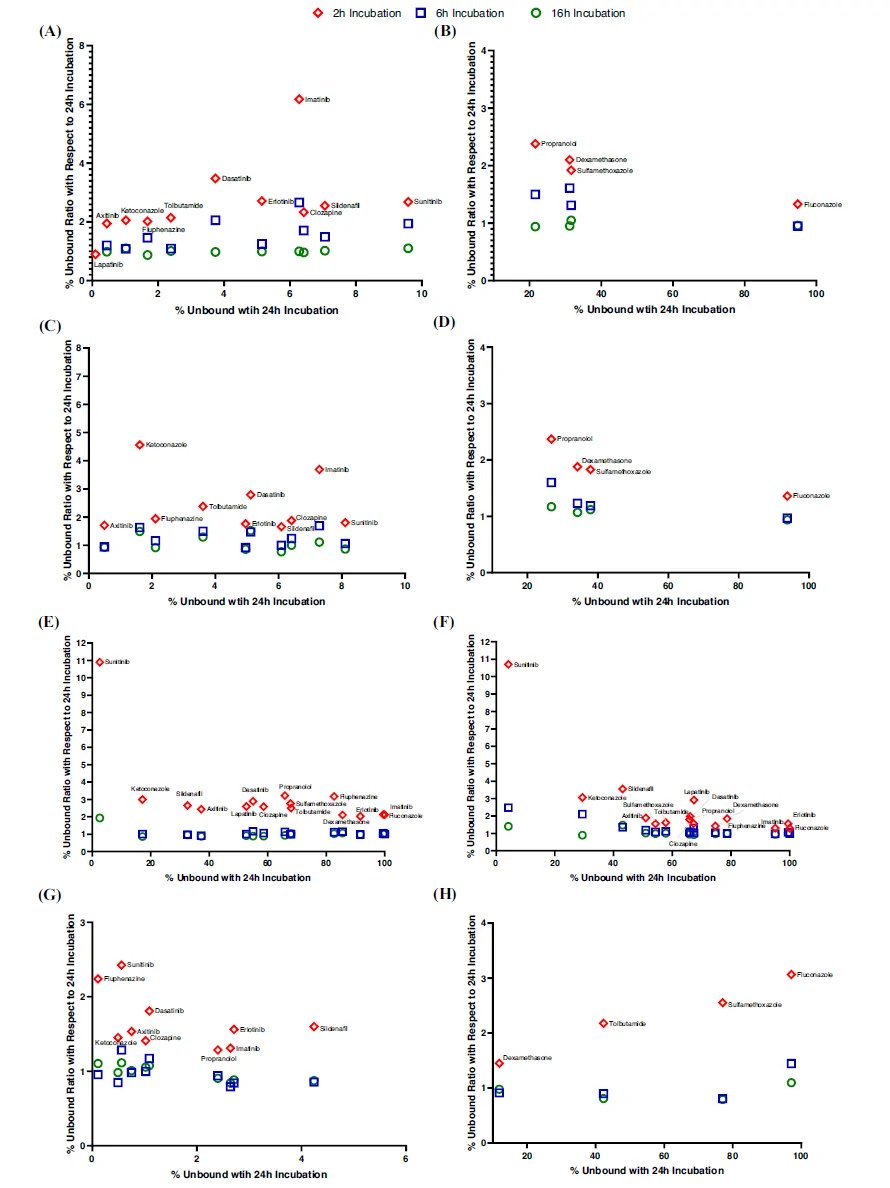
% unbound ratio at specific incubation time with respect to % unbound at the 24-hour equilibrium time. (**A**) using the RED device for <10% unbound in human plasma; (**B**) using the RED device for >10% unbound in human plasma; (**C**) using the HTD device for <10% unbound in human plasma; (**D**) using the HTD device for >10% unbound in human plasma; (**E**) using the RED device in 10% FBS; (**F**) using the HTD device in 10% FBS; (**G**) using the HTD device for <10% unbound in rat brain homogenate; (**H**) using the HTD device for >10% unbound in rat brain homogenate.

**Fig. (4):**
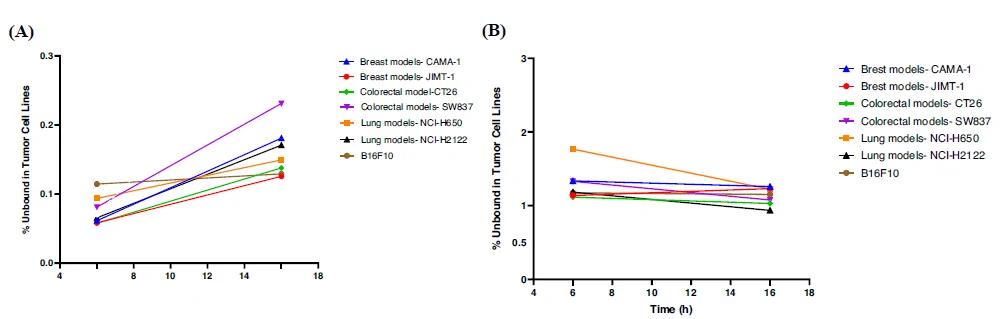
Comparison of % unbound values for nelfinavir and ritonavir in tumor cell lines. (**A**) nelfinavir at 6 hours and 16 hours; (**B**) ritonavir at 6 hours and 16 hours.

**Fig. (5):**
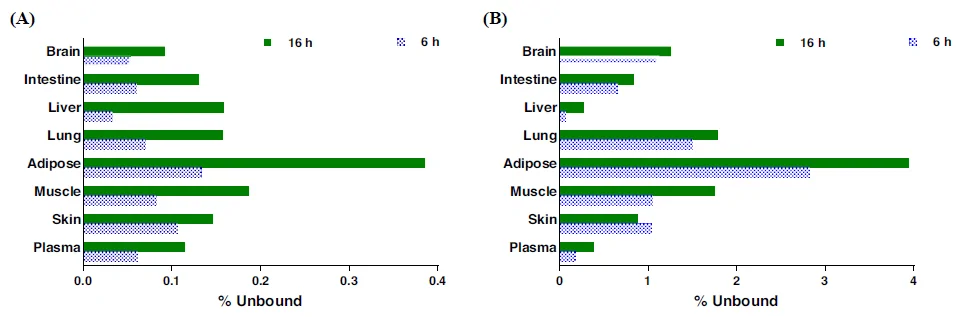
Comparison of % unbound in mouse tissues. (**A**) nelfinavir at 6 hours and 16 hours; (**B**) ritonavir at 6 hours and 16 hours.

**Table 1:** List of compounds and relevant physicochemical properties.

Compound	Structure	Molecular Weight (Free Base)	Class	pKa	LogD (pH 7.4)
Axitinib	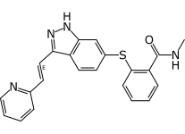	386.47	Basic	4.85 (B1), 11.93 (A1)	3.57
Clozapine	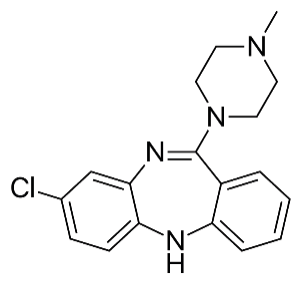	326.83	Basic	3.96 (B1), 7.62 (B2)	2.78
Dasatinib	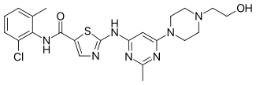	488.01	Basic	3.01 (B1), 6.93 (B2), 10.65 (A1)	2.98
Dexamethasone	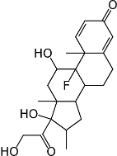	392.47	Neutral	NA	1.99
Erlotinib	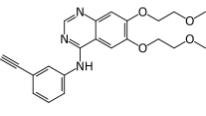	393.44	Basic	5.37 (B1)	3.21
Fluconazole	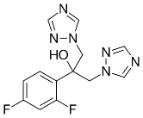	306.28	Basic	2.56 (B1), 2.94 (B2), 11.01 (A1)	0.52
Fluphenazine	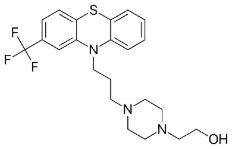	437.53	Basic	3.57 (B1), 7.63 (B2)	3. 75
Imatinib	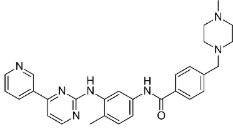	493.62	Basic	1.93 (B1), 3.16 (B2), 4.12 (B3), 7.89 (B4)	2. 42
Ketoconazole	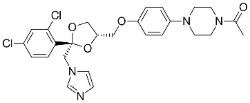	551.48	Basic	3.35 (B1), 6.38 (B2)	3.59
Lapatinib	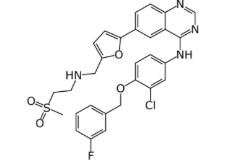	581.06	Basic	4.57 (B1), 5.87 (B2)	3.81
Propranolol	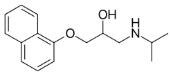	259.35	Basic	9.58 (B1)	1.18
Sildenafil	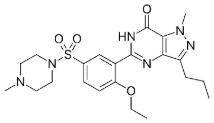	474.58	Basic	6.5 (B1), 9.2 (A1)	2.81
Sulfamethoxazole	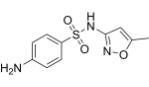	253.28	Acidic	1.55 (B1), 5.62 (A1)	-0.62
Sunitinib	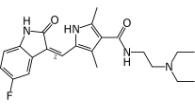	398.48	Basic	8.89 (B1)	2.43
Tolbutamide	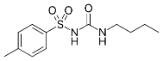	270.35	Acidic	5.52 (A1)	0.32
Nelfinavir	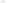 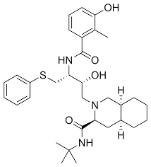	567.79	Acidic	9.32 (A1)* 8.18 (B1)*	4.72*
Ritonavir	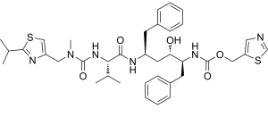	720.95	Acidic	2.84 (B1)*	5.22*

**Note:** B1, B2, B3, and B4 denote different basic sites within the same molecule; A1 denotes the first acidic site (if more than one is present). pKa for fluconazole and sildenafil were obtained from the study by Corrêa *et al*. 2012 [38] and Akula & Lakshmi 2018 [39], respectively. *Data sourced from https://go.drugbank.com.

## Data Availability

All data generated or analyzed during this study are included in this published article.

## LIST OF ABBREVIATIONS

DMSO: Dimethyl Sulfoxide
ED: Equilibrium Dialysis
FBS: Fetal Bovine Serum
HTD: High-Throughput Dialysis
IS: Internal Standard
IVIVC: *In vitro–In vivo* Correlation
LC: Liquid Chromatography
MS: Mass Spectrometry
PB: Protein Binding
PBS: Phosphate-Buffered Saline
PD: Pharmacodynamics
PK: Pharmacokinetics
PPB: Plasma Protein Binding
RED: Rapid Equilibrium Dialysis
UV: Ultraviolet
