# Supplementary material for: Optimization of Incubation Time for Accurate Protein Binding Assessment in Biological Matrices Using Equilibrium Dialysis
Source: Drug Metab Bioanal. 2026 Mar 18;19(3):297–310. doi: 10.2174/0118723128441303260129080416 (PMC13698027; doi:10.2174/0118723128441303260129080416)
Supplement: Supplementary file 1 [file DMB-19-3-297_SD1.pdf]

SUPPLEMENTARY MATERIAL

Optimization of Incubation Time for Accurate Protein Binding Assessment in Biological Matrices Using Equilibrium Dialysis

Jay Prakash Jain[1], Jianxia Shi[1], Atul Rathore[1], Austin Li[1], Chunyan Han[2], Xingjin Jiao[2] and Richard Zang[1,*]

[1]DMPK and Clinical Pharmacology, IDEAYA Biosciences, South San Francisco, CA, USA; [2]DMPK Division, Pharmaron Inc., Beijing, China

Supplemental Table 1. % unbound at different time points and ratio of each time point compared to % unbound at 24 hours in human plasma proteins using the RED or HTD devices.

Drug	RED							HTD							% unbound ratio
	% unbound				% unbound ratio			% unbound				% unbound ratio			
	2 h	6 h	16 h	24 h	24 h / 2 h	24 h / 6 h	24 h / 16 h	2 h	6 h	16 h	24 h	24 h / 2 h	24 h / 6 h	24 h / 16 h	24 h RED / 24 h HTD
Axitinib	0. 2	0. 4	0. 5	0. 4	2. 0	1. 2	1. 0	0. 3	0. 5	0. 5	0. 5	1. 7	0. 9	0. 9	0. 9
Clozapine	2. 8	3. 8	6. 7	6. 4	2. 3	1. 7	1. 0	3. 4	5. 2	6. 4	6. 4	1. 9	1. 2	1. 0	1. 0
Dasatinib	1. 1	1. 8	3. 8	3. 7	3. 5	2. 1	1. 0	1. 8	3. 5	3. 4	5. 1	2. 8	1. 5	1. 5	0. 7
Erlotinib	1. 9	4. 1	5. 2	5. 2	2. 7	1. 3	1. 0	2. 8	5. 4	5. 8	5. 0	1. 8	0. 9	0. 9	1. 0
Fluphenazine	0. 8	1. 2	1. 9	1. 7	2. 0	1. 5	0. 9	1. 1	1. 8	2. 3	2. 1	1. 9	1. 2	0. 9	0. 8
Imatinib	1. 0	2. 4	6. 3	6. 3	6. 2	2. 7	1. 0	2. 0	4. 3	6. 6	7. 3	3. 7	1. 7	1. 1	0. 9
Ketoconazole	0. 5	0. 9	0. 9	1. 0	2. 1	1. 1	1. 1	0. 4	1. 0	1. 1	1. 6	4. 6	1. 6	1. 5	0. 6
Lapatinib	0. 1	BLQ	BLQ	0. 1	0. 9	NA	NA	BLQ	0. 1	BLQ	BLQ	NA	NA	NA	NA
Sildenafil	2. 8	4. 8	6. 9	7. 1	2. 6	1. 5	1. 0	3. 7	6. 1	7. 9	6. 1	1. 7	1. 0	0. 8	1. 2
Sunitinib	3. 6	4. 9	8. 7	9. 6	2. 7	1. 9	1. 1	4. 5	7. 7	9. 4	8. 1	1. 8	1. 1	0. 9	1. 2
Tolbutamide	1. 1	2. 2	2. 4	2. 4	2. 1	1. 1	1. 0	1. 5	2. 4	2. 8	3. 6	2. 4	1. 5	1. 3	0. 7
Dexamethasone	13. 9	18. 3	31. 8	30. 2	2. 1	1. 6	1. 0	17. 2	26. 7	31. 0	33. 2	1. 9	1. 2	1. 1	0. 9
Fluconazole	70. 4	99. 0	97. 7	93. 8	1. 3	0. 9	1. 0	67. 9	95. 8	99. 0	92. 8	1. 4	1. 0	0. 9	1. 0
Propranolol	9. 1	13. 4	22. 0	20. 6	2. 4	1. 5	0. 9	11. 3	15. 7	22. 0	25. 8	2. 4	1. 6	1. 2	0. 8
Sulfamethoxazole	15. 5	23. 2	29. 2	30. 6	1. 9	1. 3	1. 1	19. 7	30. 9	32. 8	36. 9	1. 8	1. 2	1. 1	0. 8

BLQ: below the limit of quantification on the buffer side; NA: not applicable.

Note: Equilibration is assumed to be reached by 24 hours.

3117-3861/26 $65.00+.00 © 2026 Bentham Science Publishers

Supplemental Table 2: % unbound at different time points and ratio of each time point compared to % unbound at 24 hours in cellular assay medium (containing 10% FBS) using the RED or HTD devices.

Drug	RED							HTD							% un-bound ratio
	% unbound				% unbound ratio			% unbound				% unbound ratio			
	2 h	6 h	16 h	24 h	24 h / 2 h	24 h / 6 h	24 h / 16 h	2 h	6 h	16 h	24 h	24 h / 2 h	24 h / 6 h	24 h / 16 h	24 h RED / 24 h HTD
Axitinib	14. 4	39. 8	41. 8	36. 3	2. 4	0. 9	0. 9	26. 0	41. 7	49. 0	50. 0	1. 9	1. 2	1. 0	0. 7
Clozapine	21. 7	54. 4	65. 0	57. 6	2. 6	1. 1	0. 9	42. 1	49. 9	61. 5	66. 3	1. 6	1. 3	1. 1	0. 9
Dasatinib	18. 0	46. 4	61. 2	54. 0	2. 9	1. 2	0. 9	32. 3	62. 3	65. 5	65. 1	2. 0	1. 1	1. 0	0. 8
Dexamethasone	44. 1	92. 6	92. 8	90. 7	2. 0	1. 0	1. 0	62. 9	92. 6	97. 7	98. 5	1. 6	1. 1	1. 0	0. 9
Erlotinib	25. 1	73. 7	79. 4	81. 7	3. 2	1. 1	1. 0	51. 4	70. 3	75. 2	73. 7	1. 4	1. 1	1. 0	1. 1
Fluconazole	46. 1	99. 0	93. 3	99. 0	2. 1	1. 0	1. 1	76. 1	99. 0	99. 0	99. 0	1. 3	1. 0	1. 0	1. 0
Fluphenazine	5. 8	16. 2	18. 9	16. 2	3. 0	1. 0	0. 9	9. 6	13.9	31. 6	28. 4	3. 1	2. 1	0. 9	0. 6
Imatinib	19. 3	51. 8	56. 7	51. 7	2. 6	1. 0	0. 9	22. 0	64. 9	71. 4	66. 4	2. 9	1. 0	0. 9	0. 8
Ketoconazole	12. 3	32. 5	33. 2	31. 6	2. 7	1. 0	1. 0	12. 1	30. 8	28. 2	42. 1	3. 6	1. 4	1. 5	0. 8
Lapatinib	0. 2	BLQ	1. 4	2. 6	10. 9	NA	1. 9	0. 4	1. 7	3. 0	4. 2	10. 7	2. 5	1. 4	0. 6
Propranolol	26. 2	67. 0	65. 3	67. 0	2. 5	1. 0	1. 0	34. 7	50. 8	56. 2	56. 8	1. 6	1. 1	1. 0	1. 2
Sildenafil	39. 9	73. 7	79. 4	84. 6	2. 1	1. 2	1. 1	41. 6	78. 0	77. 4	77. 7	1. 9	1. 0	1. 0	1. 1
Sulfamethoxa-zole	45. 7	94. 2	96. 9	98. 5	2. 1	1. 0	1. 0	71. 4	95. 7	99. 0	94. 1	1. 3	1. 0	1. 0	1. 1
Sunitinib	19. 5	57. 4	70. 1	64. 9	3. 2	1. 1	0. 9	35. 6	58. 9	68. 4	64. 9	1. 8	1. 1	1. 0	1. 0
Tolbutamide	23. 5	66. 9	66. 6	66. 8	2. 8	1. 0	1. 0	34. 0	49. 5	53. 8	53. 3	1. 6	1. 1	1. 0	1. 3

BLQ: below the limit of quantification on the buffer side; NA: not applicable.

Note: Equilibration is assumed to be reached by 24 hours.

Supplemental Table 3. % unbound at different time points and ratio of each time point compared to % unbound at 24 hours in rat brain homogenate using the HTD device.

Drug	HTD						
	% unbound				% unbound ratio		
	2 h	6 h	16 h	24 h	24 h / 2 h	24 h / 6 h	24 h / 16 h
Axitinib	0. 5	0. 8	0. 8	0. 8	1. 5	1. 0	1. 0
Clozapine	0. 7	1. 0	1. 0	1. 0	1. 4	1. 0	1. 1
Dasatinib	0. 6	0. 9	1. 0	1. 1	1. 8	1. 2	1. 1
Erlotinib	1. 7	3. 2	3. 1	2. 7	1. 6	0. 8	0. 9
Fluphenazine	0. 1	0. 1	0. 1	0. 1	2. 2	1. 0	1. 1
Imatinib	2. 0	3. 3	3. 1	2. 6	1. 3	0. 8	0. 8
Ketoconazole	0. 3	0. 6	0. 5	0. 5	1. 5	0. 9	1. 0

Lapatinib	BLQ	BLQ	BLQ	0. 0	NA	NA	NA
Sildenafil	2. 7	5. 0	4. 9	4. 2	1. 6	0. 9	0. 9
Sunitinib	0. 2	0. 4	0. 5	0. 6	2. 4	1. 3	1. 1
Tolbutamide	18. 4	46. 2	51. 3	41. 3	2. 2	0. 9	0. 8
Dexamethasone	8. 2		12. 1	11. 9	1. 5	0. 9	1. 0
Fluconazole	30. 7	66. 2	87. 5	96. 1	3. 1	1. 5	1. 1
Propranolol	1. 9	2. 6	2. 7	2. 4	1. 3	0. 9	0. 9
Sulfamethoxazol	29. 2	94. 6	96. 2	76. 1	2. 6	0. 8	0. 8

BLQ: below the limit of quantification on the buffer side; NA: not applicable.

Note: Equilibration is assumed to be reached by 24 hours.

Supplemental Table 4: % unbound of 1 uM of nelfinavir and ritonavir at 6 and 16 hours and their ratios in tumor cell lines.

Drug	Tumor models	Test concentration (µM)	Dilution factor	% unbound	% unbound	% unbound ratio
				6 h	16 h	16 h /6 h
Nelfinavir	Breast models-CAMA-1	1	4	0. 06	0. 18	2. 95
Nelfinavir	Breast models-JIMT-1	1	4	0. 06	0. 13	2. 16
Nelfinavir	Colorectal models-CT26	1	4	0. 06	0. 14	2. 39
Nelfinavir	Colorectal models-SW837	1	4	0. 08	0. 23	2. 85
Nelfinavir	Lung models-NCI-H650	1	4	0. 09	0. 15	1. 60
Nelfinavir	Lung models-NCI-H2122	1	4	0. 07	0. 17	2. 60
Nelfinavir	B16F10	1	4	0. 11	0. 13	1. 13
Ritonavir	Breast models-CAMA-1	1	4	1. 34	1. 26	0. 94
Ritonavir	Breast models-JIMT-1	1	4	1. 14	1. 23	1. 08
Ritonavir	Colorectal models-CT26	1	4	1. 12	1. 03	0. 92
Ritonavir	Colorectal models-SW837	1	4	1. 33	1. 08	0. 81
Ritonavir	Lung models-NCI-H650	1	4	1. 77	1. 22	0. 69
Ritonavir	Lung models-NCI-H2122	1	4	1. 18	0. 94	0. 79
Ritonavir	B16F10	1	4	1. 17	1. 15	0. 98

Supplemental Table 5: % unbound of 1 uM nelfinavir and ritonavir at 6 and 16 hours and their ratios in mouse matrices.

Drug	Tissues	Test concentra-tion (µM)	Dilution factor	% unbound	% unbound	% unbound ratio
				6 h	16 h	16 h /6 h
Nelfinavir	Brain	1	4	0. 05	0. 09	1. 74
Nelfinavir	Intestine	1	8	0. 06	0. 13	2. 17
Nelfinavir	Liver	1	4	0. 03	0. 16	4. 76
Nelfinavir	Lung	1	8	0. 07	0. 16	2. 23
Nelfinavir	Adipose	1	4	0. 13	0. 39	2. 89

Nelfinavir	Muscle	1	10	0. 08	0. 19	2. 23
Nelfinavir	Skin	1	8	0. 11	0. 15	1. 38
Nelfinavir	Plasma	1	1	0. 06	0. 11	1. 87
Ritonavir	Brain	1	4	1. 13	1. 26	1. 12
Ritonavir	Intestine	1	8	0. 65	0. 84	1. 29
Ritonavir	Liver	1	4	0. 07	0. 27	3. 72
Ritonavir	Lung	1	8	1. 50	1. 79	1. 19
Ritonavir	Adipose	1	4	2. 83	3. 94	1. 39
Ritonavir	Muscle	1	10	1. 05	1. 75	1. 66
Ritonavir	Skin	1	8	1. 04	0. 89	0. 85
Ritonavir	Plasma	1	1	0. 18	0. 38	2. 09
